# Sleep Apnea Classification Algorithm Development Using a Machine-Learning Framework and Bag-of-Features Derived from Electrocardiogram Spectrograms

**DOI:** 10.3390/jcm11010192

**Published:** 2021-12-30

**Authors:** Cheng-Yu Lin, Yi-Wen Wang, Febryan Setiawan, Nguyen Thi Hoang Trang, Che-Wei Lin

**Affiliations:** 1 Department of Otolaryngology, National Cheng Kung University Hospital, College of Medicine, National Cheng Kung University, Tainan 704, Taiwan; yu621109@ms48.hinet.net; 2 Department of Environmental and Occupational Medicine, National Cheng Kung University Hospital, College of Medicine, National Cheng Kung University, Tainan 704, Taiwan; 3 Sleep Medicine Center, National Cheng Kung University Hospital, College of Medicine, National Cheng Kung University, Tainan 704, Taiwan; 4 Department of Biomedical Engineering, College of Engineering, National Cheng Kung University, Tainan 701, Taiwan; yiwen.wtmh@gmail.com (Y.-W.W.); febryans2802@gs.ncku.edu.tw (F.S.); hoangtrangnguyen181@gmail.com (N.T.H.T.); 5 Medical Device Innovation Center, National Cheng Kung University, Tainan 704, Taiwan; 6 Institute of Gerontology, College of Medicine, National Cheng Kung University, Tainan 701, Taiwan

**Keywords:** sleep apnea, time–frequency transformation, bag-of-features, support vector machine, k-nearest neighbor algorithm, ensemble learning

## Abstract

Background: Heart rate variability (HRV) and electrocardiogram (ECG)-derived respiration (EDR) have been used to detect sleep apnea (SA) for decades. The present study proposes an SA-detection algorithm using a machine-learning framework and bag-of-features (BoF) derived from an ECG spectrogram. Methods: This study was verified using overnight ECG recordings from 83 subjects with an average apnea–hypopnea index (AHI) 29.63 (/h) derived from the Physionet Apnea-ECG and National Cheng Kung University Hospital Sleep Center database. The study used signal preprocessing to filter noise and artifacts, ECG time–frequency transformation using continuous wavelet transform (CWT), BoF feature generation, machine-learning classification using support vector machine (SVM), ensemble learning (EL), k-nearest neighbor (KNN) classification, and cross-validation. The time length of the spectrogram was set as 10 and 60 s to examine the required minimum spectrogram window time length to achieve satisfactory accuracy. Specific frequency bands of 0.1–50, 8–50, 0.8–10, and 0–0.8 Hz were also extracted to generate the BoF to determine the band frequency best suited for SA detection. Results: The five-fold cross-validation accuracy using the BoF derived from the ECG spectrogram with 10 and 60 s time windows were 90.5% and 91.4% for the 0.1–50 Hz and 8–50 Hz frequency bands, respectively. Conclusion: An SA-detection algorithm utilizing BoF and a machine-learning framework was successfully developed in this study with satisfactory classification accuracy and high temporal resolution.

## 1. Introduction

Sleep apnea (SA) is a sleep disorder with high prevalence, particularly among middle-aged and elderly subjects. SA prevalence in the overall population ranges from 9% to 38% [1]. Frost and Sullivan (2016) estimated the annual medical cost of undiagnosed SA among U.S. adults to be nearly USD 149.6 billion per year [2]. To reduce the number of undiagnosed SA patients, sleep examinations with fewer channels of physiological signals, such as type III home sleep testing (HST), have received increasing attention in recent years. Facco et al. demonstrated that HST has relatively high intraclass correlation and unconditional agreement with in-lab polysomnography (PSG) testing in SA diagnosis [3]. Dalewski et al. estimated the usefulness of employing modified Mallampati scores (MMP) and the upper airway volume (UAV) to diagnose obstructive SA among patients with breathing-related sleep disorders compared to the more expensive and time-consuming PSG [4]. Philip et al. showed that self-reported sleepiness at the wheel is a better predictor than the apnea hypopnea index (AHI) for sleepiness-related accidents among obstructive SA patients [5]. Furthermore, Kukwa et al. determined that there was no significant difference in the percentage of supine sleep between in-lab PSG and HST. However, women presented more supine sleep with HST than with PSG [6]. Thus, the development of sleep technologies such as type III or type IV monitors may be beneficial for the development of sleep medicines due to the convenience of such medicines compared to PSG.

SA episodes are generally accompanied by abnormal breathing [7] and relative sympathetic nervous system hyperactivity [8]. Several studies attempted to utilize electrocardiogram (ECG) technology to develop automatic SA-detection algorithms since ECG can yield both electrocardiogram-derived respiration (EDR), representing breathing activity [9], and heart rate variability (HRV), representing autonomous nervous function indexes [10]. Most existing automatic SA-detection algorithms using ECG can be categorized into EDR-, HRV-, and cardiopulmonary coupling (CPC)-related approaches.

Many studies have shown that EDR is correlated with respiratory variation [11,12,13,14]. EDR is used to estimate respiration based on changes in the morphology of the ECG. Varon et al. proposed a method to detect SA by using an EDR signal derived from a single-lead ECG [12]. In this study, the ECG was transformed into EDR signals in three different ways; moreover, two novel feature sets were proposed (principal components for QRS complexes and orthogonal subspace projections between respiration and heart rate) and compared with the two most popular features in heart rate variability analysis. The accuracy was 85% for the discrimination of both apnea and hypopneas together. An algorithm based on deep learning approaches for automatically extracting features and detecting SA events in an EDR signal was proposed by Steenkiste et al. [14]. The authors employed a balanced bootstrapping scheme to extract efficient respiratory information and trained long short-term memory (LSTM) networks to produce a robust and accurate model.

Several studies have investigated the association between HRV and SA, and many SA-detection algorithms were developed using the HRV parameter [15,16,17,18,19,20]. HRV assesses the variability in periods between consecutive heartbeats, which change under the control of the autonomic nervous system. Quiceno-Manrique et al. [16] transformed an HRV signal into the time–frequency domain using short-time Fourier transform (STFT) and extracted indices including spectral centroids, spectral centroid energy, and cepstral coefficients to detect SA with 92.67% accuracy. However, these approaches required the collection of at least 3 min of ECG signals to include low-frequency components. Martin-Gonzalez et al. [19] developed a detection algorithm using machine-learning methods to characterize and classify SA based on an HRV feature selection process, focusing on the underlying process from a cardiac-rate point of view. The authors generated linear and nonlinear variables such as Cepstrum coefficients (CCs), Filterbanks (Fbank), and detrended fluctuation analysis (DFA). This algorithm achieved 84.76% accuracy, 81.45% sensitivity, 86.82% specificity, and 0.87 area under the receiver operating characteristic (ROC) curve AUC value. Singh et al. [20] implemented a convolutional neural network (CNN) using a pre-trained AlexNet model in order to improve the detection performance of obstructive SA based on a single-lead ECG scalogram with accuracy of 86.22%, sensitivity of 90%, specificity of 83.8%, and an AUC value of 0.8810.

One of the classic analysis tools for SA disorders is electrocardiogram-derived CPC sleep spectrograms using RR interval and EDR coupling characteristics. Thomas et al. [21] used CPC to evaluate ECG-based cardiopulmonary interactions against standard sleep staging among 35 PSG test subjects (including 15 healthy subjects). Spectrogram features included normal-to-normal sinus inter-beat interval series and corresponding EDR signals. However, using the kappa statistic, agreement with standard sleep staging was poor, with 62.7% for the training set and 43.9% for the testing set. Meanwhile, the cyclic alternating pattern scoring was higher, with 74% for the training set and 77.3% for the testing set. Guo et al. [22] found that CPC high frequency coupling (HFC) proportionally reduced sleep disorder behavior and that HFC durations were negatively correlated with the nasal-flow-derived respiratory disturbance index. Liu et al. [23] developed a CPC method based on the Hilbert–Huang transform (HHT) and found that HHT-CPC spectra provided better temporal and frequency resolution (8 s and 0.001 Hz, respectively) compared to the original CPC (8.5 min and 0.004 Hz, respectively).

Previous studies showed that HRV, EDR, and CPC features can be used to develop automated SA-classification algorithms. However, the RR interval and EDR need a relatively longer time to collect the data; hence, the temporal resolution is poor for algorithms based on HRV or EDR features. Sleep CPC is a good visualization tool for RR interval and EDR coupling since it can indicate sleep quality or sleep-based breathing disorders. However, CPC’s temporal resolution for sleep is also poor, as breathing- disorder patterns present large variance, making automated classification difficult. Thus, developing an automated SA algorithm with high temporal resolution was taken as the main research aim of the present study. In this work, we used ECG spectrogram features to develop an algorithm that employs bag-of-features (BoF) techniques and machine-learning classifiers to identify various patterns in SA episodes from ECG spectrograms. Penzel et al. [24] initially exhibited different patterns on the ECG time–frequency spectrogram between normal and SA episodes. However, the authors did not develop automatic classification for SA episodes. Thus, the aim of this study was to develop automatic SA classification based on ECG time–frequency spectrograms.

## 2. Materials and Methods

### 2.1. Sleep Apnea ECG Database

Two datasets, the National Cheng Kung University Hospital Sleep Center Apnea Database (NCKUHSCAD) and Physionet Apnea-ECG Database [25] (PAED), were used in this study. NCKUHSCAD was used to observe differences in the apnea and normal periods of the ECG spectrogram. PAED was used to validate the proposed algorithm, as PAED is a public database, which enabled us to compare our results with the existing literature. NCKUHSCAD included information collected from patients who underwent overnight PSG in the Sleep Center of NCKU Hospital (Taiwan) between December 2016 and August 2018. Patients with the following conditions were excluded: PSG recordings for continuous positive airway pressure ventilation titration, the use of hypnotic medicine during the test, and missing data. The study protocol was approved by the Institutional Review Board of NCKUH (protocol number: B-ER-108-426). The database included 50 recordings sampled at 200 Hz, with annotations provided for 10 and 60 s as either normal breathing, hypopnea, or apnea disordered breathing. Hypopnea was defined as a ≥30% reduction in baseline airflow for at least 10 seconds combined with either arousal in an electroencephalogram for ≥3 seconds or oxygen desaturation ≥ 3%. Apnea was defined as a ≥90% decrease in airflow over a 10-second period with concomitant respiratory-related chest wall movement for obstructive apnea [26]. In this study, ECG recordings with hypopnea and apnea annotations were merged together as the apnea group.

The 50 recordings from NCKUHSCAD were rearranged into three groups (APEG-A, APEG-B, and APEG-C) (APEG is the abbreviation of APnEa Group) to fulfill different purposes during algorithm performance evaluation.

- NCKUHSCAD-APEG-A included 11 participants who provided severe SA recordings (30 < AHI ≤ 45), with an average ± standard deviation AHI of 39.25 ± 5.78/h.
- NCKUHSCAD-APEG-B included 35 participants who suffered from SA (AHI ≥ 10), with an average ± standard deviation AHI of 39.83 ± 23.08/h.
- NCKUHSCAD-APEG-C included the whole database, with an average ± standard deviation AHI of 29.02 ± 25.49/h.

PAED [25] was adopted to develop and verify the proposed algorithm’s performance. The ECG recordings of PAED were sampled at 100 Hz, and sleep recording durations ranged between 7 and 10 h depending on the participant. The database included 35 recordings, with SA annotations provided on a minute-by-minute basis—i.e., each minute of ECG recording was annotated as either N (normal breathing) or A (disordered breathing including the occurrence of an apnea episode).

PAED included three participant groups: A: apnea, B: borderline, and C: healthy (the control), with 20, 5, and 10 participants, respectively. Those in Group A were known to be related to people definitely suffering from obstructive SA and had total apnea durations > 100 min for each recording. The range of ages among subjects in this group was 38–63, and the AHI of this group’s subjects ranged between 21 and 83; those in Group B were borderline and had total apnea episode durations of 10–96 min. The age range within this group was 42–53, and the AHI ranged between 0 and 25; those in Group C had no obstructive SA or very low levels of disease and total apnea durations between 0 and 3 min [27]. Recordings b05 from Group B and c05 from Group C were excluded because recording b05 contained a grinding noise, and c05 was identical to c06. Therefore, only 33 recordings were ultimately included in this study [28].

The remaining 33 recordings from the PAED [28] were also regrouped into three categories:

- PAED-APEG-A included 8 participants with severe SA recordings from group A (30 < AHI ≤ 45, average ± standard deviation AHI: 39.14 ± 3.60/h, age 51.38 ± 6.43 years, and weight 87.88 ± 9.42 kg).
- PAED-APEG-B included participants who suffered from SA—i.e., all of group A (21 < AHI < 83) and group B (0 < AHI < 25, except b05). Thus, APEG-B included 1 female and 23 males, with an average ± standard deviation AHI of 41.55 ± 23.45/h, an age of 51.42 ± 6.50 years, and a weight of 93.04 ± 16.67 kg.
- PAED-APEG-C included the whole database (excluding b05 and c05). Thus, APEG-C included 4 females and 29 males, with an average ± standard deviation AHI of 30.23 ± 27.35/h, an age of 46.85 ± 9.80 years, and a weight of 86.67 ± 18.23 kg. This group featured the same arrangement of participants used in [12,16,17,18,20,29,30].

Table 1 presents the ECG spectrogram results counted for the different groups after dividing the nocturnal ECG signals into 1 min time windows. The ECG signals were divided into 1 min time window because the sleep expert labelled the ECG signal event (normal vs. apnea) based on a 1 min time window, and some previous studies also employed this database from PhysioNet and used 1 min time window divisions to separate ECG signal events [12,17,18,19]. Contaminated window ECG spectrograms in PAED (but not in NCKUHSCAD) were removed, which was also done in [12,14,16], since raw ECG signals could contain a wide range of noise caused by the patient’s movement, poor patch contact, electrical interference, measurement noise, or other disturbances.

### 2.2. Sleep Apnea Detection Algorithm Using a Machine-Learning Framework and Bag-of-Features Derived from ECG Spectrograms

**Figure 1:**
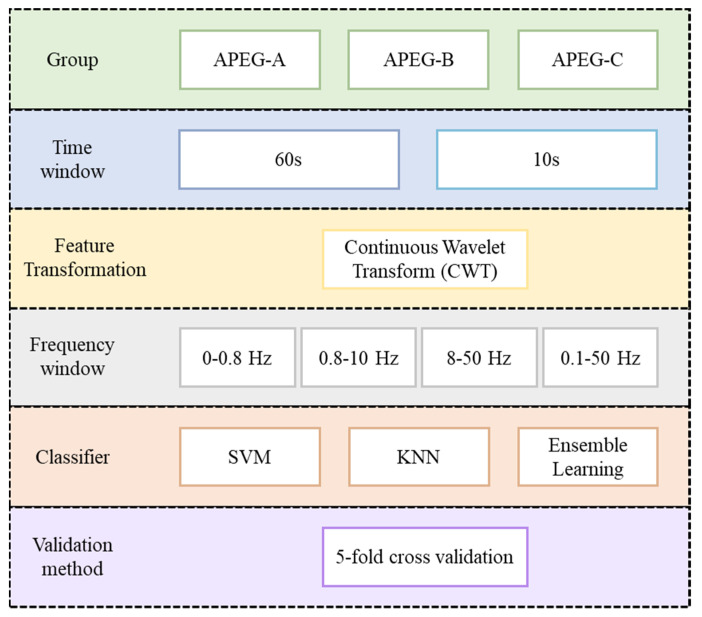
Proposed sleep apnea detection algorithm using machine learning framework and bag -of-features derived from ECG spectrograms (SVM: Support Vector Machine; KNN: k-nearest neighbor).

This study proposes an SA-detection algorithm using a machine-learning framework and BoF derived from ECG spectral intensity differences between SA and normal breathing. Figure 1 shows the proposed algorithm flowchart. Single-lead ECG data were input and then divided into consecutive 60 s ECG windows. The time–domain ECG for each window was transformed into the time–frequency spectrogram to obtain ECG spectrograms as the main feature. The BoF technique was then used to obtain features to discriminate ECG spectrograms for SA and normal breathing. Finally, machine-learning classifiers, including support vector machine (SVM), ensemble learning (EL), k-nearest neighbor (KNN), and cross-validation were used to obtain the classification results.

### 2.3. Data Preprocessing

In this study, data preprocessing consisted of zero means computation and windowing preprocessing parameters. In this method, the zero-means subtract the mean from the ECG signals to eliminate trend-variation effects, and then nocturnal ECG spectra are segmented into consecutive 60 s windows (to match the database annotation), where windows with large noise are excluded for algorithm development. The PAED [25,28] and NKCUHSCAD utilized in this study labeled every 60 s window as containing an apnea episode or not.

### 2.4. Time–Frequency Transformation of ECG

The time–domain ECG for each window after data preprocessing was transformed into the time–frequency domain to facilitate better SA-episode classification. Continuous wavelet transform (CWT) [31] was used due to its high-resolution time–frequency components. CWT uses different time lengths to adaptively optimize the resolution in different frequency ranges. A CWT wavelet is a small wave compared to a sinusoidal wave—i.e., a brief oscillation that can be dilated or shifted according to the input signal. Common wavelet types include Meyer, Morlet, and Mexican hat. We selected the Morlet wavelet regime for this study to specify the extraction of several prominent frequency bands. Thus, the CWT regime can be expressed as

$$X_{w}\left(s,\tau \right)=\frac{1}{\sqrt{s}}\overset{\infty }{\underset{-\infty }{\int }}x\left(t\right)\psi^{*}\left(\frac{t-\tau }{s}\right)\;dt$$

where $x\left(t\right)\in L^{2}\left(\mathbb{R}\right)$ is a time series function, $\psi^{*}\left(t\right)$ is the wavelet function, and $s\in {\mathbb{R}}^{+}\;(s>0)$ is a scaling or dilation factor.

**Figure 2:**
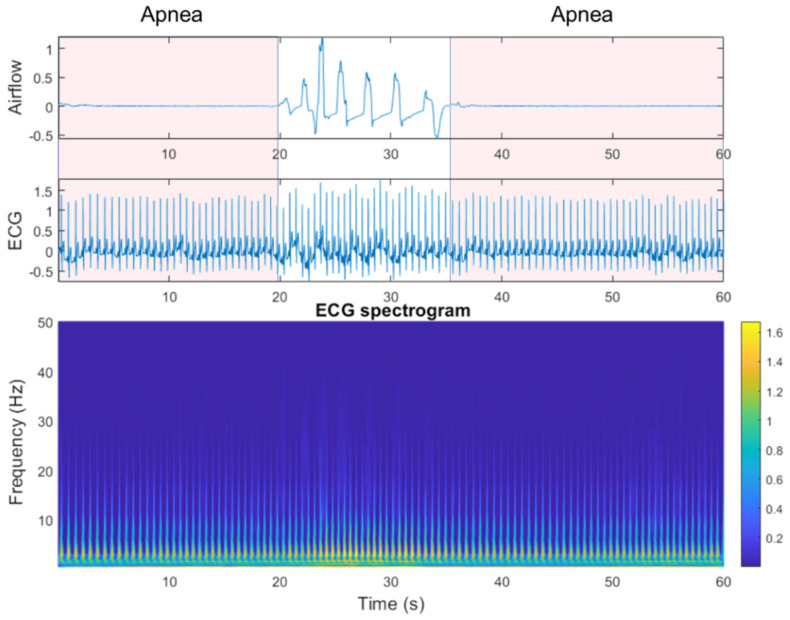
Airflow, electrocardiogram (ECG), and ECG spectrograms from apnea to non-apnea, followed by apnea again.

To observe the differences between SA episodes and normal breathing (i.e., periods with no SA episode), we used clinical data from the National Cheng Kung University Hospital sleep center for ECG spectrogram observation. Figure 2 shows example benchmark data that include an SA episode, followed by a return to normal breathing and then a subsequent SA episode. ECG spectrogram differences between apnea onset and normal breathing, derived by continuous wavelet transform (CWT), were significant. The power spectrum intensity for normal breathing was much stronger than that for SA episodes in the 5–10 Hz band.

**Figure 3:**
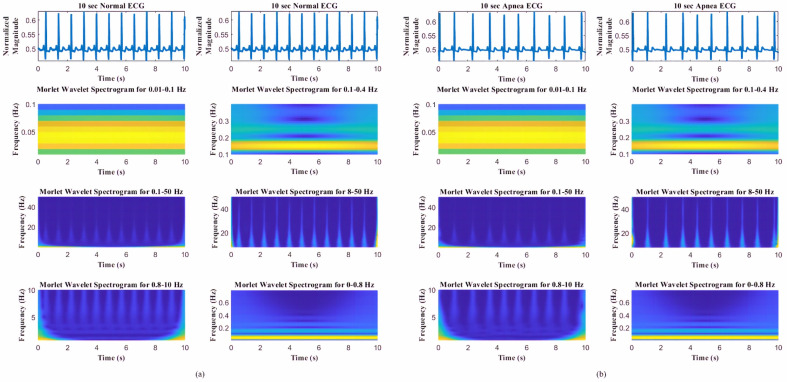
Spectrograms of different frequency bands (0.01–0.1, 0.1–0.4, 0.5–50, 8–50, 0.8–10, and 0–0.8 Hz) [17,18,25] for (**a**) normal ECG and (**b**) apnea ECG events.

To achieve high-temporal-resolution pattern visualization, the different spectrogram frequency bands were extracted to classify SA based on other authors’ observations and findings [21,22,31]: (1) overall frequency 0.1–50 Hz, (2) high frequency 8–50 Hz, (3) middle frequency 0.8–10 Hz, and (4) low frequency 0–0.8 Hz. Thomas et al. [21,32] and Guo et al. [22] verified that certain frequency bands were associated with periodic respiration during sleep-disorder breathing (0.01–0.1 Hz), as well as physiologic respiratory sinus arrhythmia and deep sleep (0.1–0.4 Hz). As shown in Figure 3, the frequency range definitions of Thomas et al. and Guo et al. do not offer good pattern visualization features between normal ECG and apnea ECG events.

### 2.5. Feature Extraction Using Bag-of-Features

**Figure 4:**
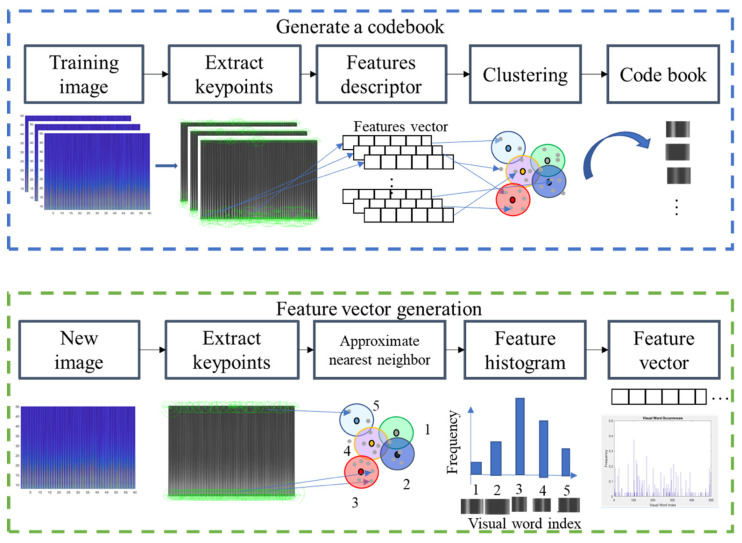
Codebook and feature vector generation for the proposed bag-of-features method.

To extract features that best discriminate the spectrogram with apnea onset and normal breathing, we used the bag-of-features (BoF) or bag-of-visual-words [33], a visual classification approach commonly employed for image classification. The BoF corresponds to the frequency histogram of a particular image pattern occurrence in a given image and was also successfully used for text classification. Figure 4 shows the BoF flowchart used in this study.

The two main steps in this process were codebook generation and BoF feature-vector extraction. Codebook generation extracted representative features (visual words) to describe an image. ECG spectrograms were then regarded as images, with visual words being generated as follows. Key points (points of interest) were extracted from training images using a speeded-up robust features (SURF) detector [34], and 64-dimensional descriptors were used to describe the key points. Descriptors were then clustered by k-means clustering, and the resulting clusters were compacted and subsequently separated based on similar characteristics. Each cluster center represented a visual word (analogous to vocabulary), thereby producing a codebook (analogous to a dictionary) [33]. The codebook was then used to obtain the BoF for each ECG spectrogram image. Then, image key points were extracted, and image descriptors were obtained. Here, each descriptor corresponded to the closest visual word, and visual word occurrences in an image were counted to produce a histogram representing the image. Figure 4 shows how these feature vectors were then directly input to machine-learning classifiers for SA classification.

**Figure 5:**
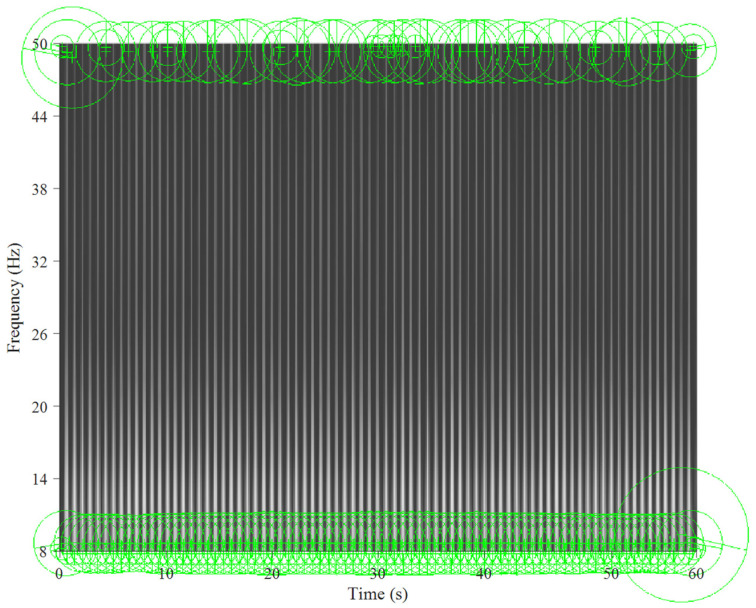
Key points extracted from an ECG spectrogram of normal breathing for a 60 s window and 8–50 Hz band.

**Figure 6:**
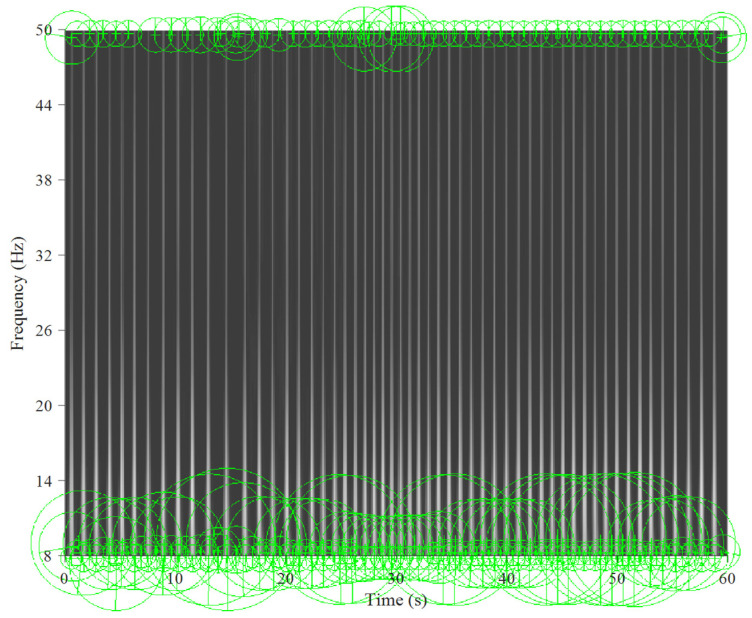
Key points extracted from the sleep apnea ECG spectrogram for a 60 s window and 8–50 Hz band.

Figure 5 and Figure 6 show the key points automatically extracted by the BoF in the ECG spectrogram algorithm for normal breathing and SA, respectively. Each green circle represents a key point able to best discriminate breathing patterns for each ECG spectrogram. Key points were similar for normal breathing but varied significantly for SA spectrograms.

### 2.6. Machine-Learning classifiers

Support vector machine (SVM) is a supervised learning model for classification and regression. The core concept of SVM is to find the hyperplane that best separates data into two classes. In binary classification, generally, p-dimensional data can be separated by a (p-1)-dimensional hyperplane. The best hyperplane is defined as that with the largest margin between the two classes, and the closest point on the hyperplane boundary is considered the support vector [35].

The *k*-nearest neighbor (KNN) classifier is a simple supervised learning method and uses the nearest distance approach in deciding the group of the new data in the training set. During the training phase, the feature space is split into several regions and the training data are mapped into the similar groups of feature space. The unlabeled testing data are then classified into a certain group of feature space based on the minimum distance. Distance is an important factor for the model and can be determined, e.g., by Euclidean, Mahalanobis, and cosine-distance metrics [36,37].

Ensemble learning (EL) combines multiple learning algorithms and weight sets to construct a better classifier model. Prediction using an ensemble algorithm requires extensive computation compared to using only a single model. Therefore, ensembles can compensate for poor learning algorithms by performing extra computations. EL methods generally use fast algorithms, although slower algorithms can also benefit from ensemble techniques. Many different ensemble models have been proposed, including the Bayes optimal classifier and boosting and bootstrap aggregating (bagging) techniques [38]. EL bagged trees and subspace KNN were employed in this study. The bagged trees method established a set of decision tree models trained on randomly selected portion of data; then, the predictions are merged to achieve final predictions using averaging [38]. Meanwhile, subspace KNN was designed using majority vote rule, where the random subspace ensemble method was used with nearest neighbor learner type of 30 learners [39,40].

### 2.7. k-Fold Cross-Validation

*k*-fold cross-validation is a well-developed validation technique [41]. The first step is to divide the data samples into *k* subgroups. Subsequently, each subgroup can be selected as the testing set with the remaining (*k*-1) subgroups as the training set. In this way, *k*-fold cross-validation repeats training and testing *k* times, and the final accuracy is the average of the *k* accuracy values for each iteration. In this study, the *k*-fold cross-validation with *k* = 5 was performed at the spectrogram-level instead of the participant-level, as some studies have done [16,42,43,44]. As evaluation criteria, the accuracy, sensitivity, and specificity parameters were computed for classification performance metrics. The definitions of these metrics can be found in Table 2 [45]. 

## 3. Experimental Results

The experiments were executed using MATLAB R2020a software on several computers with 24 GB installed RAM, Intel® Core™ i5-8400 CPU @2.80 GHz, and NVIDIA GeForce GTX 1060 6 GB mounted graphic card. The Classification Learner toolbox from MATLAB was utilized to perform the machine-learning classification. Hyperparameter optimization was performed using nested five-fold cross-validation and grid search using a 60 s time window based on the best frequency range, 8~50 Hz, and machine-learning model construction, SVM (see Table 3). Table 4 and Table 5 compare the selected APEGs from NCKUHSCAD and PAED for the frequency bands (overall frequency, 0.1–50 Hz; high frequency, 8–50 Hz; middle frequency, 0.8–10 Hz; and low frequency, 0–0.8 Hz) along with SVM, KNN, and EL classifiers using a time-window length of 60 s and five-fold validation. The best classification accuracy for NCKUHSCAD-APEG-A, NCKUSCAD-APEG-B, and NCKUHSCAD-APEG-C was 84.4%, 80.8%, and 83.8% at 8–50 Hz, 8–50 Hz, and 8–50 Hz, respectively, using SVM, EL, and SVM. Conversely, the best classification accuracy for PAED-APEG-A, PAED-APEG-B, and PAED-APEG-C was 88.2%, 88.3%, and 91.4% at 8–50, 8–50, and 8–50 Hz using SVM, EL, and EL, respectively. The PAED-APEG-C classification accuracy for the 0.8–10 Hz band using EL was 89.1%, which was only 2.3% less than the best accuracy (91.4%). Hence, we selected the 8–50 Hz band and EL classifier to classify all SA episodes in the remaining work.

SA detection in a short time window was an important consideration for this study. Therefore, we also investigated a shorter window length (10 s). For example, eleven subjects from NCKUHSCAD-APEG-A were selected, and different frequency bands were compared via five-fold cross-validation. The time window length was too short to provide variation in the lower frequency band; hence, that band was not considered in this comparison. Table 6 shows that the 10 s time window provided greater accuracy than the 60 s time window, with the best accuracy reaching 95% for the 8–50 Hz band using SVM.

## 4. Discussion

To the best of our knowledge, our study is the first to use an ECG spectrogram for SA detection. This proposed algorithm used high-temporal resolution feature visualization of the ECG spectrogram in differentiating normal and SA breathing. Using this high-temporal resolution pattern visualization, the proposed algorithm was able to achieve high accuracy, sensitivity, and specificity in SA detection.

### 4.1. ECG Variation during Rapid Eye Movement (REM) and Non-REM Sleep Stages

Sleep is a dynamic situation of consciousness characterized by rapid changes in autonomic activity that regulates coronary artery tone, systemic blood pressure, and heart rate. In the analysis of 24-hour heart rate variability (HRV), a nocturnal increase in the standard deviation of mean RR intervals commonly occurrs. In the study by Zemaityte et al. [46] and Raetz et al. [47], they observed that, when compared to the wakefulness stage, non-REM sleep stage was associated with lower overall HRV and during REM sleep stage, the opposite phenomenon was observed, an increase in overall HRV. Otherwise, some studies examined the highest nocturnal activity during REM sleep due to the peripheral sympathetic nerve activity with no difference in heart rates between the sleep stages [48,49,50].

In this study, by investigating the REM and non-REM partitions on the SA classification using PAED 60 s time window based on the best frequency range (8~50 Hz) and machine-learning model (SVM), it was concluded that the ECG variation (HRV) did not significantly affect the SA classification performance. Both REM and non-REM partitions of imbalanced and balanced datasets generated for PAED-APEG-A, PAED-APEG-B, and PAED-APEG-C groups presented a good classification performance with the average accuracy was up to 81.42% for REM stage and 79.2% for non-REM stage (see Table 7). The balancing data were randomly performed in order to address more imbalanced apnea and normal events as a consequence of partitioning the datasets into REM and non-REM.

### 4.2. Per Subject Classification (Leave-One-Subject-Out Cross-Validation)

A more realistic scenario to the medical application, which is required to classify a new unseen subject into the model, is the so-called per-subject classification. Leave-one-subject-out cross-validation (LOSOCV) was used in this study to perform the per-subject classification. In LOSOCV, one subject is set aside for the evaluation (testing) and the model is trained on remaining subjects. The process is repeated each time with a different subject for evaluation and results are averaged over all folds (subjects).

Results from k-fold cross-validation PAED 60 s time window based on 8~50 Hz frequency range and SVM classifier experimental setting demonstrates that the model almost certainly can detect subjects with disease if training and testing sets are not separated in terms of subjects, leaving data related to a subject in both sets. As the result shows, it can achieve a high level of accuracy (89.13%). With all other experimental settings remaining the same, except PAED-APEG-C group as some subjects in the Group C were excluded since they did not experience anapnea event, when the LOSOCV was applied the accuracy decreased significantly to 70% (Table 8, Table 9 and Table 10). This probably means that in the case of k-fold cross-validation where subject data are in both training and testing sets, the algorithm is learning the subject rather than the disease condition.

### 4.3. Performance Comparison with the Existing Literature

Table 11 compares the proposed algorithm with various current best-practice algorithms from the literature using PAED. Quinceno-Manrique et al. [16], Nguyen et al. [17], Sannino et al. [18], and Hassan [29] used HRV-extracted features in the time domain and frequency domain along with non-linear methods, such as features from spectral centroids, spectral centroid energy, recurrence statistics, and wavelet transform, while Varon et al. [12] used EDR as the feature (84.74% accuracy, 84.71% sensitivity, and 84.69% specificity). Singh et al. [20] proposed a CNN-based deep learning approach using the time–frequency scalogram transformation of an ECG signal (86.22% accuracy, 90% sensitivity, and 83.8% specificity). Surrel et al. [30] used RR intervals and RS amplitude series (85.70% accuracy, 81.40% sensitivity, and 88.40% specificity). The proposed method achieved significantly better performance in accuracy, sensitivity, and specificity compared to all other considered methods for 1 min time windows and also achieved comparable accuracy to the compared methods (90.5%) for the 10 s time windows, indicating that the proposed method offers higher temporal resolution.

Quinceno-Manrique et al. (89.02% accuracy) [16], Nguyen et al. (85.26% accuracy, 86.37% sensitivity, and 83.47% specificity) [17], Sannino et al. (85.76% accuracy, 65.82% sensitivity, and 66.03% specificity) [18], Hassan (87.33% accuracy, 81.99% sensitivity, and 90.72% specificity) [29], and Surrel et al. [30] used HRV features to analyze ECG signals, which had two major disadvantages. First, the HRV frequency band was low, meaning that the time windows had to be extended to ensure that signal variation was properly expressed (e.g., Quinceno-Manrique et al. used 3 min time windows, and HRV features required 5 min windows [16]). OSA onsets and offsets could occur several times within windows of these lengths, significantly limiting the practical applications of these approaches. Second, HRV features use simplified information from the original ECG (QRS complexes), so considerable physiological information, such as ECG signal morphology, could be lost. The same situation occurs for EDR [12], as EDR was derived from the R-wave amplitude, which also used simplified ECG information. Conversely, the proposed algorithm directly used ECG spectrograms to classify SA and normal breathing. Hence, the main advantage of the proposed method is the identification of significant variations in the occurrence of apnea episodes.

Similar to the proposed method, Singh et al. [20] also employed time–frequency transformation using CWT to obtain the scalogram of an ECG signal. The deep learning layers of CNN-pre-trained AlexNet were used for feature extraction. At the final layer, the decision fusion of some machine-learning approaches (SVM, KNN, EL, and Linear Discriminant Analysis (LDA)) was utilized for classification. However, the main difference and advantage of this study algorithm is the application of a more sophisticated time–frequency transformation using the Morlet wavelet in order to observe significant variations between several frequency ranges and obtain high temporal resolution. Moreover, the use of bag-of-features in the proposed method enables robust features called SURFs to be generated and outperformed the Singh et al. study performance with 91.4% accuracy and 92.4% specificity.

### 4.4. Limitations and Future Developments

Although the proposed algorithm exhibited excellent performance, there were several limitations in this study. First, a limited sample size from PAED was used to validate the proposed algorithm. Second, in PAED, insufficient physiological data and subject disease history was available for further analysis since such information was not provided. Mass data collection from the Sleep Center NCKUH could be a solution for these drawbacks. Third, the proposed algorithm could not be applied to patients with cardiovascular disease complications since their ECG spectrograms tended to be somewhat irregular and not affected only by SA.

Future works could identify physiological meanings for the OSA features automatically extracted from the AI algorithm, test a large-scale group of participants from the sleep center database, and develop an algorithm to discriminate SA and cardiovascular disease using ECG data.

## 5. Conclusions

This paper proposed a new algorithm to classify SA patterns from ECG spectrograms. Four different frequency bands were considered along with three classifiers. High accuracy was obtained when applying time–frequency spectrograms to SA, and the features extracted by visual classification revealed previously unknown physiological significance, such that SA detection was feasible.

The algorithm provided superior accuracy compared to current common-practice approaches over generally shorter time windows (60 s) compared to those used in earlier models. Acceptable accuracy was also derived for very short time windows (10 s), highlighting the considerable flexibility in potential applications for this algorithm.

## Figures and Tables

**Table 1:** General apnea group subject patterns for the proposed frequency bands.

Frequency Band	Normal Breathing Data *			Apnea Data *		
	APEG-A	APEG-B	APEG-C	APEG-A	APEG-B	APEG-C
0.1–50 Hz	4990/1778	11,236/4883	16,941/8383	1638/1799	5173/5886	5538/5895
8–50 Hz	4990/1819	11,236/4985	16,941/8665	1638/1820	5173/5902	5538/5908
0.8–10 Hz	4990/1776	11,236/4803	16,941/8568	1638/1802	5173/5775	5538/5778
0–0.8 Hz	4990/1804	11,236/5060	16,941/8526	1638/1801	5173/5931	5538/5993

* Data are given in order: algorithm performance evaluation databases NCKUHSCAD/PAED. The number before “/” is for the NCKUHSCAD dataset, and the number after “/” is for the PAED dataset.

**Table 2:** Confusion matrix and evaluation parameter equations.

Confusion Matrix		Actual Class		$\mathrm{Accuracy}=\frac{\mathrm{TP}+\mathrm{TN}}{P+N}$	$\mathrm{Specificity}=\frac{\mathrm{TN}}{\mathrm{TN}+\mathrm{FP}}$
		A	B		
Predicted Class	A	TP	FP		
	B	FN	TN	$\mathrm{Sensitivity}=\frac{\mathrm{TP}}{\mathrm{TP}+\mathrm{FN}}$	
Total		P	N		

Note: A = condition positive set; B = condition negative set; TP = true positive; FP = false positive; FN = false negative; TN = true negative; P = TP + FN; N = FP + TN.

**Table 3:** Nested five-fold cross-validation training and validation performance using a 60 s time window based on 8~50 Hz frequency range and SVM classifier of PAED and NCKUHSCAD.

Database	Accuracy (%)			Sensitivity (%)			Specificity (%)		
	APEG-A	APEG-B	APEG-C	APEG-A	APEG-B	APEG-C	APEG-A	APEG-B	APEG-C
PAED	87.35	88.06	90.43	89.70	90.40	88.72	85.01	85.32	91.55
NCKUHSCAD	83.40	80.15	83.54	56.78	63.48	57.17	92.14	87.82	92.16

**Table 4:** Five-fold cross-validation classification performance using a 60 s time window for NCKUHSCAD.

Frequency Band	Accuracy (%)			Sensitivity (%)			Specificity (%)		
	APEG-A	APEG-B	APEG-C	APEG-A	APEG-B	APEG-C	APEG-A	APEG-B	APEG-C
SVM									
0.1–50 Hz	81.6	77.7	81	50.2	45.4	32.4	91.9	92.5	96.9
8–50 Hz	84.4 ^#^	80.5	83.8 ^#^	57.3	60.6	51.6	93.3	89.6	94.3
0.8–10 Hz	83.3	79.6	82.6	53.8	56.6	42.9	93	90.1	95.5
0–0.8 Hz	79.9	70.9	76.8	18.9	22	6	100	93.4	100
KNN									
0.1–50 Hz	81	77.8	81.6	49.6	57	47.1	91.3	87.3	92.8
8–50 Hz	83.6	79.3	82.6	56.7	62.5	54.6	92.4	87.1	91.7
0.8–10 Hz	82.7	78	81.3	52.4	53.7	44.6	92.6	89.1	93.4
0–0.8 Hz	78.8	69.9	76.3	29.6	11.4	9	95	96.8	98.2
EL									
0.1–50 Hz	82.2	78.4	82.5	60.2	61.1	52.2	89.4	86.3	92.4
8–50 Hz	84	80.8 ^#^	83.7	63.7	70.6	65.3	90.6	85.5	89.7
0.8–10 Hz	82.5	78.6	82	60.5	60.8	48.8	89.7	86.8	92.9
0–0.8 Hz	78.8	70.8	76.6	41.1	36.5	20.5	91.2	86.6	95

Note: ^#^ denotes the highest accuracy in APEG-A/APEG-B/APEG-C.

**Table 5:** Five-fold cross-validation classification performance using a 60 s time window for PAED.

Frequency Band	Accuracy (%)			Sensitivity (%)			Specificity (%)		
	APEG-A	APEG-B	APEG-C	APEG-A	APEG-B	APEG-C	APEG-A	APEG-B	APEG-C
SVM									
0.1–50 Hz	77.2	82.8	86.5	78.8	81.3	79.1	75.5	84.5	91.3
8–50 Hz	88.2 ^#^	88.3	90.9	90.1	90.7	89.1	86.3	85.6	92.1
0.8–10 Hz	88.2	87.8	90.1	89.8	90.9	87.2	86.7	84.2	92.1
0–0.8 Hz	65.7	68.0	71.4	63.5	73.4	52.4	68.0	61.7	83.8
KNN									
0.1–50 Hz	75.2	81.5	84.9	66.8	77.2	72.2	83.5	86.4	93.3
8–50 Hz	86.1	86.5	89.5	82.3	85.4	82.6	90.0	87.7	94.0
0.8–10 Hz	88.0	86.1	88.5	84.3	83.6	79.7	91.8	89.1	94.3
0–0.8 Hz	59.3	62.5	66.0	52.1	62.1	35.0	66.5	62.9	86.5
EL									
0.1–50 Hz	75.7	81.8	85.6	71.1	81.9	78.0	80.2	81.8	90.7
8–50 Hz	86.9	88.3 ^#^	91.4 ^#^	88.9	90.7	89.8	84.8	85.5	92.4
0.8–10 Hz	87.2	86.5	89.1	87.8	88.6	85.5	86.5	84.0	91.4
0–0.8 Hz	65.2	69.5	69.5	64.7	45.9	45.9	65.8	85.1	85.1

Note: ^#^ denotes the highest accuracy in APEG-A/APEG-B/APEG-C.

**Table 6:** Five-fold cross-validation classification performance using a 10 s time window for NCKUHSCAD.

Frequency Band	Accuracy (%)			Sensitivity (%)			Specificity (%)		
	APEG-A	APEG-B	APEG-C	APEG-A	APEG-B	APEG-C	APEG-A	APEG-B	APEG-C
SVM									
0.1–50 Hz	92.7	93.2	93.9	79.4	80.4	77.4	97	97.4	97.5
8–50 Hz	95 ^#^	93.3 ^#^	94.9 ^#^	84.3	79.2	77.7	98.5	97.8	98.6
0.8–10 Hz	80.1	81.5	85.9	38	40.3	37.3	93.8	94.8	96.5
KNN									
0.1–50 Hz	81.7	84.2	87.3	45.8	53.5	45.5	93.4	94	96.5
8–50 Hz	87.1	85.1	88.2	59.2	52.4	47.3	96.3	95.6	97.1
0.8–10 Hz	76.8	79	84.2	10.8	36	28.8	98.3	92.9	96.2
EL									
0.1–50 Hz	91.3	91.9	92.8	70.5	73.3	64.1	98	97.9	99
8–50 Hz	94	91.8	93.3	79.6	78.4	66.8	98.7	96.1	99.1
0.8–10 Hz	79.3	80.8	85.3	29.6	35.3	29.1	95.5	95.4	97.5

Note: ^#^ denotes the highest accuracy in APEG-A/APEG-B/APEG-C.

**Table 7:** The REM and Non-REM classification performance using a PAED 60 s time window based on 8~50 Hz frequency range and SVM classifier.

Sleep Stage	Accuracy (%)			Sensitivity (%)			Specificity (%)		
	APEG-A	APEG-B	APEG-C	APEG-A	APEG-B	APEG-C	APEG-A	APEG-B	APEG-C
Imbalanced Dataset									
REM	83.1	78.7	75	90.5	78.5	84.7	61.3	78.8	59.1
Non-REM	74.8	81.4	77.8	72.3	81.6	78.7	77.1	81.1	77
Balanced Dataset									
REM	89.6	78.7	83.4	90	78.5	85.3	89.2	78.8	81.5
Non-REM	75.5	86.6	79.1	74.5	86.8	80.8	76.4	86.4	77.5

**Table 8:** Leave-one-subject-out cross-validation classification performance using a PAED-APEG-A 60 s time window based on 8~50 Hz frequency range and SVM classifier.

Evaluation Parameter	APEG-A Subject								Average
	a03	a05	a08	a13	a16	a17	a19	a20	
Accuracy	75.0	54.3	76.5	85.2	73.3	67.2	80.2	52.1	70.48
Sensitivity	98.6	70.1	57.8	77.4	68.4	95.7	66.5	26.4	70.11
Specificity	54.7	32.4	88.4	92.7	83.1	49.5	88.8	93.0	72.83

**Table 9:** Leave-one-subject-out cross-validation classification performance using a PAED-APEG-B 60 s time window based on 8~50 Hz frequency range and SVM classifier.

**Evaluation** **Parameter**	**APEG-B Subject**												
	**a01**	**a02**	**a03**	**a04**	**a05**	**a06**	**a07**	**a08**	**a09**	**a10**	**a11**	**a12**	**a13**
Accuracy	92.35	20.83	84.17	58.54	81.90	65.42	72.71	75.83	83.75	77.08	65.95	52.08	81.46
Sensitivity	100	0.26	93.24	56.69	91.80	28.96	85.37	59.36	86.89	87.95	32.97	52.85	65.11
Specificity	21.05	100	76.36	79.49	68.18	87.88	52.69	86.35	73.68	74.81	91.18	43.90	97.14
**Evaluation Parameter**	**APEG-B Subject**											**Average**	
	**a14**	**a15**	**a16**	**a17**	**a18**	**a19**	**a20**	**b01**	**b02**	**b03**	**b04**		
Accuracy	81.88	75	76.04	78.06	84.38	84.79	73.13	90.63	47.92	63.57	28.81	70.68	
Sensitivity	99.74	80.62	72.50	93.48	89.04	68.11	65.08	10.53	88.17	84.62	90.0	70.14	
Specificity	11.34	58.87	83.13	68.47	45.10	95.25	85.95	93.93	38.24	59.72	27.32	67.50	

**Table 10:** Leave-one-subject-out cross-validation classification performance using a PAED-APEG-C 60 s time window based on 8~50 Hz frequency range and SVM classifier.

**Evaluation** **Parameter**	**APEG-C Subject**												
	**a01**	**a02**	**a03**	**a04**	**a05**	**a06**	**a07**	**a08**	**a09**	**a10**	**a11**	**a12**	**a13**
Accuracy	89.80	21.46	83.96	69.17	76.90	64.58	70.83	78.54	79.58	76.25	57.38	44.79	81.25
Sensitivity	94.92	1.0	77.48	67.80	77.05	16.39	85.71	61.50	78.69	79.52	2.75	43.96	65.96
Specificity	42.11	100	89.53	84.62	76.70	94.28	47.31	89.42	82.46	75.57	99.16	53.66	95.92
**Evaluation Parameter**	**APEG-C Subject**												
	**a14**	**a15**	**a16**	**a17**	**a18**	**a19**	**a20**	**b01**	**b02**	**b03**	**b04**	**c02**	**c06**
Accuracy	81.46	73.96	73.33	78.61	88.75	78.96	56.25	93.96	63.54	72.38	41.67	98.54	91.90
Sensitivity	98.43	78.93	70.31	65.94	92.31	57.84	38.98	0	74.19	70.77	50.0	0	0
Specificity	14.43	59.68	79.38	86.49	58.82	92.20	83.78	97.83	60.98	72.68	41.46	98.75	92.12
**Evaluation Parameter**	**APEG-C Subject**			**Average**									
	**c07**	**c09**	**c10**										
Accuracy	95.48	90.71	47.86	73.17									
Sensitivity	25.0	0	0	50.88									
Specificity	96.15	91.15	47.97	76.02									

**Table 11:** Proposed algorithm comparison with current best-practice algorithms.

Author (Year)	Database (Population)	Time-Window Length	Method	Accuracy (%)	Sensitivity (%)	Specificity (%)
Lin et al. (this paper)	Physionet Apnea-ECG (APEG-A: 3660 min, APEG-B: 11,160 min, APEG-C *: 15,180 min)	1 min	CWT + SVM/KNN/EL	91.4	89.8	92.4
Quinceno-Manrique et al. [16] (2009)	Physionet Apnea-ECG (all observations: 8928 intervals, best observations: 4000 intervals)	3 min	SPWVD + PCA + KNN	89.02	Not mentioned	Not mentioned
Nguyen et al. [17] (2014)	Physionet Apnea-ECG (whole database: Group A, B, and C)	1 min	RQA + greedy forward feature selection + SVM and neural network	85.26	86.37	83.47
Sannino et al. [18] (2014)	Physionet Apnea-ECG (whole database: Group A, B, and C)	1 min	Frequency domain, time domain, and non-linear parameters + DEREx	85.76	65.82	66.03
Varon et al. [12] (2015)	Physionet Apnea-ECG (34,324 annotated min)	1 min	EDR (Ramp/PCA/kPCA) + LS-SVM	84.74	84.71	84.69
Hassan [29] (2016)	Physionet Apnea-ECG (whole database: Group A, B, and C)	1 min	TQWT + NIG + AdaBoost	87.33	81.99	90.72
Surrel et al. [30] (2018)	Physionet Apnea-ECG (34,313 recorded min)	1 min	Apnea scoring (energy) + SVM	85.70	81.40	88.40
Singh et al. [20] (2019)	Physionet Apnea-ECG (whole database: Group A, B, and C)	1 min	CWT + AlexNet CNN + Decision Fusion (SVM, KNN, Ensemble, LDA)	86.22	90	83.8

Note: CWT: continuous wavelet transform; SVM: support vector machine; KNN: *k*-nearest neighbor; EL: ensemble learning; TQWT: tunable-Q factor wavelet transform; NIG: normal inverse gaussian; AdaBoost: adaptive boosting; SPWVD: smoothed pseudo Wigner–Ville distribution; PCA: principal component analysis; RQA: Recurrence Quantification Analysis; DEREx: Differential Evolution-based Rule Extractor; EDR: ECG derived respiration; Ramp: R-peak amplitude; kPCA: kernel principal component analysis; LS-SVM: least-squares support vector machine. * The APEG-C classification result was chosen because this dataset was comparable with the existing literature, consisting of the whole PhysioNet database (Groups A, B, and C).

## Data Availability

The Physionet Apnea-ECG Database (PAED) is openly available at Physionet at https://doi.org/10.13026/C23W2R (accessed on 15 October 2021). However, the National Cheng Kung University Hospital Sleep Center Apnea Database (NCKUHSCAD) is not publicly available due to privacy and ethical issues.
